# Unraveling the Molecular Mechanisms of the Neurodevelopmental Consequences of Fetal Protein Deficiency: Insights From Rodent Models and Public Health Implications

**DOI:** 10.1016/j.bpsgos.2024.100339

**Published:** 2024-06-03

**Authors:** Pieter Vancamp, Morgane Frapin, Patricia Parnet, Valérie Amarger

**Affiliations:** a Nantes Université, Institut National de Recherche pour l'Agriculture, l'alimentation et l'Environnement, UMR1280, Physiopathologie des Adaptations Nutritionnelles, l'Institut des Maladies de l'Appareil Digestif, Nantes, France; b Organismal and Evolutionary Biology Research Programme, Faculty of Biological and Environmental Sciences, University of Helsinki, Helsinki, Finland

**Keywords:** Cognitive potential, Fetal brain development, Maternal protein intake, Neuropsychiatric disorders, Protein deficiency, Rodent model

## Abstract

Fetal brain development requires increased maternal protein intake to ensure that offspring reach their optimal cognitive potential in infancy and adulthood. While protein deficiency remains a prevalent issue in developing countries, it is also reemerging in Western societies due to the growing adoption of plant-based diets, some of which are monotonous and may fail to provide sufficient amino acids crucial for the brain’s critical developmental phase. Confounding variables in human nutritional research have impeded our understanding of the precise impact of protein deficiency on fetal neurodevelopment, as well as its implications for childhood neurocognitive performance. Moreover, it remains unclear whether such deficiency could predispose to mental health problems in adulthood, mirroring observations in individuals exposed to prenatal famine. In this review, we sought to evaluate mechanistic data derived from rodent models, placing special emphasis on the involvement of neuroendocrine axes, the influence of sex and timing, epigenetic modifications, and cellular metabolism. Despite notable progress, critical knowledge gaps remain, including understanding the long-term reversibility of effects due to fetal protein restriction and the interplay between genetic predisposition and environmental factors. Enhancing our understanding of the precise mechanisms that connect prenatal nutrition to brain development in future research endeavors can be significantly advanced by integrating multiomics approaches and utilizing additional alternative models such as nonhuman primates. Furthermore, it is crucial to investigate potential interventions aimed at alleviating adverse outcomes. Ultimately, this research has profound implications for guiding public health strategies aimed at raising awareness about the crucial role of optimal maternal nutrition in supporting fetal neurodevelopment.

## Plain Language Summary

The Developmental Origins of Health and Disease theory posits that suboptimal conditions during early life exert a profound influence on adult health, potentially predisposing individuals to conditions such as neuropsychiatric disorders. By reviewing studies in rodents, we identified common mechanisms of how inadequate fetal protein uptake alters brain development and may contribute to anxiety, impaired memory function, and altered metabolism in adulthood. Adequate protein consumption during pregnancy is therefore critical to support healthy brain development.

The developmental origins of health and disease theory embodies the idea that suboptimal environmental conditions in early life predispose to chronic noncommunicable diseases in adulthood, such as cancer, cardiometabolic disease, and neuropsychiatric disorders (1). An illustrative case is the cohort of individuals born to mothers who were pregnant during the Dutch famine of 1944 to 1945. At the age of 50 to 60 years, they performed worse on cognitive tests and were more likely to develop schizophrenia (2, 3, 4), an outcome that bore striking resemblance to a wider demographic of adults living in Anhui province, China, who experienced famine after the spring of 1959 (5). Nutritional deficiency that arises from either a caloric deficit or inadequate dietary nutrients remains a prevalent environmental stressor for the fetus and a major cause of intrauterine growth restriction (IUGR). IUGR, characterized by fetal weight below the 10th percentile for gestational age (6), represents a clinical condition with established links to delayed intellectual development (7) and increased susceptibility to mental health problems in adulthood (8).

**Figure 1:**
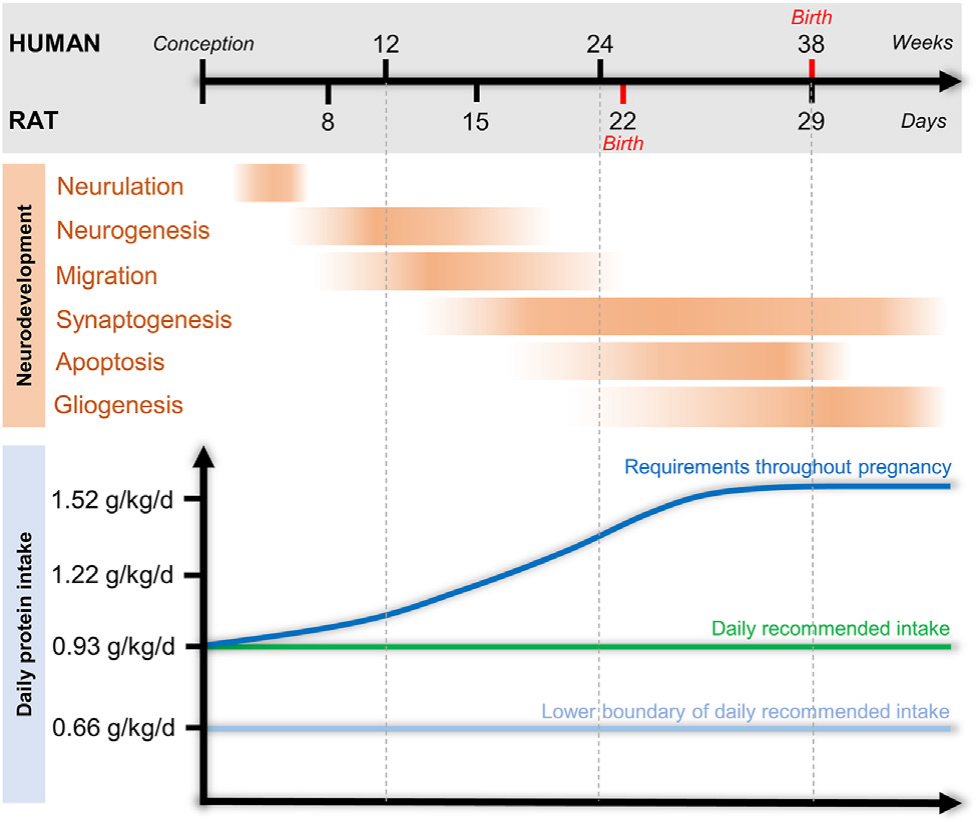
Illustration of key developmental events in rat and human brains, highlighting protein intake requirements for human fetal development. Rodents, chosen for their ease of use, shorter life span, and well-documented dietary needs, serve as valuable models. However, it is crucial to note their comparatively premature birth compared with humans. A newborn rat mirrors a human fetus at the end of the second trimester, while a human newborn aligns with a rat at postnatal day 7 (123). The period of neurogenesis in the rat spans from G10 to G20, peaking between G12 and G16, akin to the end of the first trimester to the start of the third trimester in humans. Recent advancements in protein requirement assessments during pregnancy utilizing the indicator amino acid oxidation method reveal higher needs: 1.22 to 1.52 g/kg/day at 16 and 36 weeks of pregnancy, respectively, compared with 0.93 g/kg/day in nonpregnant women (15,16). G, gestational day.

The predominant cause of IUGR in Western societies stems from fetal amino acid deficiency, primarily associated with placental dysfunction (9). Studies with primates and monochorionic twins have demonstrated that reduced amino acid transport across the placental barrier precedes the onset of IUGR (10, 11, 12). Another cause is maternal protein deficiency, a prevalent concern in low-income countries (13). However, despite improvements in staple foods since the 1970s (14), protein deficiency in pregnant women in developed countries is more pervasive than anticipated. For example, 1 in 8 pregnant women in the United States fails to meet the newly established protein requirements for the second and third trimesters of pregnancy (Figure 1) (15, 16, 17).

The resurgence of dietary protein deficiencies is multifactorial. Firstly, there is the continual rise in the adoption of plant-based diets, which are health-beneficial but often lacking in protein quantity, digestibility, and quality (18). Among the 20 amino acids, 9—histidine, lysine, methionine, phenylalanine, threonine, tryptophan, and the branched-chain amino acids (BCAAs) isoleucine, leucine, and valine—are exclusively derived from the diet. Pregnant women who adhere to monotonous plant-based and vegan diets are particularly susceptible to inadequate intake of at least some essential amino acids crucial for fetal development (19, 20, 21, 22). Another cause of recurrent amino acid deficiency is obesity, which is associated with chronic calorie-rich, unvarying diets (23). The rising incidence of bariatric surgery, especially among young women with obesity, heightens the concern about potential protein deficiency during pregnancy (24,25). Finally, the current economic landscape makes protein-rich foods, such as meat, seafood, and certain fruits and vegetables, difficult to afford for people on low incomes. A population-based Spanish study showed that pregnant women of lower socioeconomic status were more likely to have hypoproteinemia (26).

Protein accretion is crucial for optimal brain development. Amino acids function not only as the fundamental building blocks for proteins but also as precursors for neurotransmitters, hormones, and enzymes. The first 1000 days of life comprise a meticulously orchestrated sequence of events encompassing neurogenesis, migration, differentiation, synaptogenesis, apoptosis, and gliogenesis, which shape the brain’s structural foundation and set the boundaries for cognitive capacities in adulthood (Figure 1) (27). During this critical period, the brain exhibits remarkable plasticity, which places the fetus at risk of misprogramming if it is exposed to external stress such as nutritional deprivation (28, 29, 30). Confounding factors in human nutritional studies have impeded a comprehensive understanding of how protein deficiency during pregnancy affects neurodevelopment. Indirect evidence from preterm infants only suggests a correlation between fetal protein availability and childhood neurobehavioral performance (31,32), implying that a fetal deficit in protein may diminish cognitive abilities. Furthermore, it is unclear whether fetal protein deficiency may also predispose to neuropsychiatric disease in adulthood, as has been observed on multiple occasions in individuals who experienced acute prenatal caloric restriction (Table 1) (4,5,33).

**Table 1:** Exemplary Studies of the Impact of Gestational Malnutrition or Famine in Nonhuman Primates and Humans, Respectively, on Neurodevelopment, Cognitive Performance, and Associated Susceptibility to Neuropsychiatric Disorders in Adulthood

Analyzed Subjects	Neurodevelopmental and Behavioral Phenotype	Source
Epidemiological/Field Studies		
595 71- to 74-Year-Old Men and Women Exposed vs. Nonexposed to Prenatal Famine^a^	Self-perceived cognitive problems ↑ in women exposed during late gestation and ↑ in men exposed during early gestation Consultancies with health care practitioner for cognitive issues ↑	(125)
297 56- to 59-Year-Old Men and Women Exposed to Prenatal Famine vs. 737 Healthy Participants^a^	Selective attention abilities ↓	(3)
9 Patients With Schizophrenia vs. 9 Healthy Participants Exposed to Prenatal Famine, and 9 vs. 9 Not Exposed to Prenatal Famine^a^	Intracranial volume ↓ in patients with schizophrenia exposed to prenatal famine than those without exposure	(126)
6417 ≥45-Year-Old Adults Exposed to Prenatal Famine vs. Nonexposed Individuals^b^	Cognitive status, word recall, visuospatial episodic memory ↓	(127)
People Diagnosed With Schizophrenia (*n* = 4597) From 1971 to 2001^b^ vs. Total Population and Birth Records	Adjusted risk of schizophrenia of those born during famine ↑	(5)
83 Mothers With CZS-Affected Children	% of mothers with CZS-affected children ↑ when low-protein intake (protein intake ref = women between 19 and 59 years)	(112)
130 Women in 4 Rural Guatemala Villages	Reading, knowledge numeracy at 22–29 years ↑ and educational achievement ↑ when completed primary school for those who received extra protein (6.4 g/100 mL) during prenatal period and first 2 years	(128)
Baboon Studies		
Baboons at 90% Term Following Exposure to 30% MNR From 16% to 90% of Gestation	At 90% of gestation: HPA axis overactivation due to ↑ plasma cortisol and ACTH NPY ↑, GR ↑, pGR ↑, POMC ↓, pSTAT3 ↓ Brain serotonergic expression ↓ and neurons ↓ IGF and mTOR system ↓ Perturbated neurodevelopmental processes and metabolism	(129, 130, 131, 132, 133)
Juvenile Baboons Born to MNR Mothers (30% Caloric Restriction, Gestation + Lactation)	At 3.3 years of age (before onset of sexual maturity): Motivation ↓ Associative learning in males ↓, in females ↑ Accuracy working memory performance ↓, impulsivity ↑	(134)

ACTH, adrenocorticotropic hormone; CZS, congenital Zika-virus syndrome; GR, glucocorticoid receptor; HPA, hypothalamus-pituitary-adrenal; IGF, insulin-like growth factor; MNR, maternal nutrient restriction; mTOR, mechanistic target of rapamycin; NPY, neuropeptide Y; pGR: phosphorylated glucocorticoid receptor; POMC, pro-opiomelanocortin; pSTAT3: phosphorylated STAT3; STAT3, signal transducer and activator of transcription 3.

a Dutch famine (1944–1945).

b Chinese famine (1959–1961).

To address how fetal protein deficiency may lead to adverse neurological outcomes, we sought to review mechanistic data derived from rodent models, with an emphasis on the involvement of neuroendocrine axes, the influence of sex and timing, and epigenetic modifications and cellular metabolism, before proposing perspectives on how the field can move forward.

## Neuroanatomical and Behavioral Consequences

Efforts to evaluate the impact of a nutritional factor, or its absence, in human cohorts predominantly rely on outcomes derived from psychosocial performance and intelligence tests, which are susceptible to a multitude of nonnutritional variables, such as socioeconomic status, education, and parenting. Furthermore, the exact dietary patterns in cohorts of pregnant women and their offspring vary considerably, which impedes researchers’ ability to isolate the effects of dietary factors unequivocally. In contrast, dietary exposure in animal models can be thoroughly controlled and conducted under standardized conditions, which makes them particularly useful for nutritional research (34). Moreover, effects can be studied during embryonic, fetal, and neonatal stages. Research on brain development and behavioral end points following protein restriction has relied heavily on mouse and rat strains (Figure 1).

Most studies have employed a model of moderate protein restriction by feeding pregnant dams an isocaloric diet with 8% to 10% protein content, while control groups received a diet containing around 20% protein. More stringent models further reduced the protein content to 4% to 7% (Table 2). Others extended restriction until weaning or incorporated a period prior to conception. Low-protein diets reduce maternal amino acid levels and their transfer across the placenta to the fetus and to suckling pups during lactation (35, 36, 37). Despite hyperplasia and upregulation of amino acid transporters, the placenta is unable to restore adequate amino acid transfer to the fetus when the deficiency persists throughout gestation (38, 39, 40, 41). Consequently, the offspring have lower birth weights and an altered physiology that attempts to compensate for the sustained amino acid deficiency (42, 43, 44).

**Table 2:** Cellular and Behavioral End Points in Preclinical Rodent Studies Involving Gestational and/or Lactational Protein Restriction

Animal	PR Model	Time Window	Evaluation	Cellular Outcomes	Behavioral Outcomes	Sex	Source
SD Rats	23% vs. 9%	Gestation	P35	Impaired inhibitory network in anterior cingulate cortex, with: Microglia activation ↑ Oxidative stress ↑ Neuroinflammation ↑	NA	♂	(113)
CF1 Mice	20% vs. 8%	Gestation + lactation	8–18 weeks F1 + F2 generation	PET glucose PFC ↓ PFC transcriptome ↓ 21 dysregulated autism genes	Social motivation ↓ Recognition memory ↓	♀ and ♂	(70)
SD Rats	20% vs. 8%	Gestation	G17	Hypothalamic genes dysregulated Altered mitochondrial activity Methylation ↓ Neuronal progenitors ↑	NA	♀ and ♂	(83)
SD Rats	21% vs. 16%–18% vs. 10% vs. 5%	Gestation + lactation	6 weeks in F0, F1, and F2 generation	Dopamine ↑ Antioxidants ↓ Altered serotonin system	Reflexes ↓ Learning and memory ↓	NA	(135)
SD Rats	20% vs. 8%	Gestation + lactation Gestation or lactation	P12, P16	Hypothalamic energy pathways disrupted Dysregulation neurodevelopmental genes	NA	♂	(43)
Wistar Rats	17% vs. 6%	Gestation	P7, P14, 16 weeks	BNST neuronal dendritogenesis ↓ (nor)-epinephrine and DOPA in amygdala ↓ 11β-HSD2, GR, MR and type 1 CRH-R ↓ Corticosterone levels ↑	Anxiety-like behavior ↑	♂	(54,136)
Wistar Rats	20% vs. 10%	Gestation	P21, P97-112	5-HT_1A_ receptor function in female hippocampus ↓ Serotonergic cell bodies in the rostral raphe and 5-HT metabolism unaltered	Anxiety-like behavior ↑ Stress sensitivity ↑ Stress-induced anorexia	♀ and ♂	(59)
MF1 Mice	18% vs. 9%	Pre-implantation period (G0-G3.5) or gestation	G12.5, G14.5, G17.5, 4–20 weeks	BCAAs in uterine fluid ↓ Blastocyst megalin expression ↑ Blastocyst mTORC1 signaling ↓ NSC proliferation ↓ Neuronal differentiation and cortical thickness ↑	Anxiety-like behavior ↑	♀ and ♂	(87,91,93)
Sv129 Mice	20% vs. 8%	Gestation + lactation	10 weeks	*Egr1/2/4* genes upregulated in amygdala Downregulated *Npy1r* in amygdala of males only	Anxiety-like behavior ↑ in ♂ only	♀ and ♂	(52)
BL6 Mice	20% vs. 8%	4 weeks prior to mating + gestation + lactation	8–12 weeks	Clock genes dysregulated	Anxiety-like behavior ↑ Energy expenditure ↓ Circadian clock perturbed	♂	(49)
SD Rats	20% vs. 8%	3 weeks prior to mating + gestation	P0	*Bdnf* expression ↓ PKA/cAMP pathway activation ↓	NA	♀ and ♂	(99)
Wistar Rats	20% vs. 8%	Gestation + lactation	P90	Hippocampal *Bdnf* and *Zip268* expression ↓ Neurogenesis ↓	Learning and memory ↓ Exploration ↓	♂	(69)
African Striped Mice	19% vs. 10%	Gestation + lactation (+ after weaning or not)	8 weeks	NA	Aggressivity ↑ Anxiety-like behavior ↑ Impaired learning	♀ and ♂	(51)
SD Rats	20% vs. 8% (± MD)	3–4 weeks prior to mating + gestation + lactation	G19, P0, P21	Proliferation ↑, methylation ↓ *Dcx* ↓ *Gfap* ↑ in neural stem/progenitor cells Hippocampal expression *Insr* ↓ *Nestin* and *Igf2* ↑ at P21	NA	♀ and ♂	(98)
CF1 Mice	20% vs. 9%	5 days prior to mating + gestation + lactation	5–9 weeks	NA	Impaired social, risk, motivational, and exploratory behavior	♀ and ♂	(50)
Wistar Rats	17% vs. 6%	Gestation	16 weeks	Hippocampal CA3 dendritic atrophy Cytoarchitecture DG and CA1 unaltered	Learning and memory	♂	(137)
Wistar Rats	20% vs. 10%	Gestation + lactation	P17–22 and P90-96	Hippocampal progenitors ↓	Impaired memory Depression-like behavior	♂	(48)
Wistar Rats	20% vs. 10%	Gestation ± lactation	P90–220	Corticosterone levels ↑	Impaired learning Motivation ↓ Anxiety-like behavior ↑ in females and ↓ in males	♀ and ♂	(46,47)
Wistar Rats	20% vs. 8%	Gestation + lactation	P82–86	Cortical glutamatergic pathways, hormonal secretion and synaptic remodeling hypothalamus altered	NA	♂	(138)
Swiss Albino Mice	17% vs. 7%	6 weeks prior to mating + gestation + lactation	P7, P14, P60	Granule cells and EGL thickness ↓ Cerebellar PCs ↓	Delayed motor development	♀ and ♂	(139)
SD Rats	20% vs. 8%	Gestation + lactation	P35, P180	Hypothalamic insulin signaling and nutrient sensing ↓ Receptors and TFs in lipid metabolism 5-HT-regulated food intake ↓	Hyperphagia	♂	(140,141)
BL6 Mice	18% vs. 8.5%	Gestation + lactation	18–20 weeks	Dopamine circuitry VTA and PFC ↑ Hypomethylation *Cdkn1c* *Cdkn1c* expression ↑	Hyperactivity Altered reward processing Locomotor response to cocaine ↑	♂	(62)
Wistar Rats	20% vs. 10%	Gestation	G19	Brain lipids and DHA ↑	NA	NA	(45)
SD Rats	20% vs. 8%	Gestation (± lactation)	P0, P5, P12, P16	Impaired cell differentiation Delayed leptin surge AgRP and αMSH fibers in PVN ↓	NA	♂	(78)
Wistar Rats	20% vs. 6%	G8/G15–P28	P28	Protein levels in hippocampus and cerebral cortex ↓ BDNF and IGF-1 levels in hippocampus ↓	Impaired learning and memory	♀ and ♂	(67)
SD Rats	23.4% vs. 6%	Prenatal, postnatal, or chronic	P30, P90	Delayed GAD67 interneuron development	NA	♂	(142)
CD1 Mice	18% vs. 9%	G0–G12	G12	Brain AgRP, NPY, POMC, and LeptinR isoforms ↓	NA	NA	(82)
SD Rats	25% vs. 6%	5 weeks prior to mating + gestation	P90	Volume and neurons in hippocampal CA1 ↓ Volume pyramidal layer ↓	NA	♂	(68)
Wistar Rats	17% vs. 8%	Gestation + lactation	P20	Hypoglycemia and hypoinsulinemia VMN volume ↑ Neurons VMN and PVN ↑ NPY neurons in ARC ↓	NA	♂	(77)
Wistar Rats	20% vs. 8%	Gestation ± lactation	P90	Brain vascularization ↓	NA	♀ and ♂	(143)
Wistar Rats	20% vs. 5%	G0–G14	G15–P63	Transient ↓ neurogliogenesis, cortical thickness, synaptogenesis, and apoptosis	NA	♀ and ♂	(86)

The percentages indicate the relative protein amounts in the control and protein-restricted group diets. The studies are arranged chronologically based on their publication year, starting with the most recent.

5-HT, 5-hydroxytryptamine (serotonin); 11β-HSD2, 11-beta-hydroxysteroid dehydrogenase-like 2; AgRP, agouti-related peptide; ARC, arcuate nucleus; BCAA, branched-chain amino acid; BDNF, brain-derived neurotrophic factor; BNST, bed nucleus of the stria terminalis; CA, cornu ammonis; cAMP, cyclic adenosine monophosphate; Cdkn1c, cyclin dependent kinase inhibitor 1c; CRH-R, corticotropin-releasing hormone receptor; DG, dentate gyrus; DHA, docosahexaenoic acid; DOPA, dopamine; EGL, external germinal layer; G, gestational day; GAD67, glutamic acid decarboxylase 67; GR, glucocorticoid receptor; IGF-1, insulin-like growth factor 1; MBP, myelin basic protein; MD, methyl donor; MR, mineralocorticoid receptor; αMSH, α-melanocyte-stimulating hormone; mTORC1, mechanistic target of rapamycin complex 1; NA, not available; NPY, neuropeptide Y; NSC, neural stem cell; P, postnatal day; PC, Purkinje cell; PET, positron emission tomography; PFC, prefrontal cortex; PKA, protein kinase A; POMC, pro-opiomelanocortin; PR, protein restriction; pSTAT3, phosphorylated STAT3; PVN, paraventricular nucleus; SD, Sprague-Dawley; TF, transcription factor; VMN, ventromedial nucleus; VTA, ventral tegmental area.

The repercussions on neuropsychological performance of adult animals that were transiently exposed to a low-protein diet are diverse. A first consistent observation is the increased prevalence of anxiety-like behavior, as evidenced by various assessments such as ultrasonic vocalization after maternal separation, open-field, the elevated plus maze, and forced swim tests (45, 46, 47, 48, 49, 50, 51, 52). Anxiety is a complex behavior that is primarily governed by the limbic system—the thalamus, hypothalamus, basal ganglia, hippocampus, and amygdala—and the hypothalamus-pituitary-adrenal (HPA) axis via glucocorticoid signaling (53). Elevated corticosterone levels in adult rats gestationally exposed to protein deficiency have been reported repeatedly (45, 46, 47) and suggest alterations in HPA axis development. Juvenile rats had downregulated glucocorticoid and mineralocorticoid receptors in the amygdala (54). Individuals exposed to the Dutch famine prenatally and those born prematurely also had increased odds of experiencing anxious and depressive behavior, although no discernable impact on cortisol levels was observed in these cohorts (55, 56, 57).

Increased anxiety may also originate from the improper development of limbic regions and subsequent miscommunication within or between them. In rats, gestational protein deficiency impaired neuronal differentiation and dendritogenesis in the bed nucleus of the stria terminalis (45), a small structure downstream of the amygdala known for its involvement in anxiety and addiction in humans (58). In the amygdala, Nätt *et al.* found that decreased *Npy1r* (neuropeptide Y receptor 1) messenger RNA due to the suppressive effect of early growth response factors (e.g., EGR1) may be responsible for increased anxiety in male rats (52). Another plausible explanation for this behavioral anomaly is impaired serotonergic signaling, which is strongly implicated in anxiety. Maternal protein deficiency reduced substrate affinity for the serotonin 5-HT_1A_ receptor in the hippocampus of adult female offspring (59). This observation echoes findings from experiments conducted in the 1990s that showed an altered hippocampal neuronal circuitry due to impaired serotonergic signaling (60,61). Additionally, gestational and lactational protein deficiency induced overexpression of dopamine-related genes and increased numbers of tyrosine hydroxylase and dopaminergic neurons in the ventral tegmental area and prefrontal cortex, respectively, in adult mice. This increased the dopaminergic-driven locomotor response to cocaine and caused hyperactive behavior (62). Collectively, the data suggest that developmental protein deficiency can adversely impact the HPA axis, limbic system, and higher brain structures to such an extent that it lowers the threshold for anxious behavior later in life. However, the relative contribution of each component remains unclear.

Learning and memory impairments have also been observed consistently across studies, suggesting dysfunction of the hippocampus and higher cortical regions. The latest neuroimaging techniques in humans revealed that fetal undernutrition reduces gray and white matter volume (63), which are correlated with life-long neurobehavior alterations (64). Optimal gray matter configuration hinges on a precisely timed transition between cycling neural stem cells, expanding the progenitor pool, and their subsequent exit from the cell cycle to contribute to neuronal differentiation (65). Consequently, premature cell cycle exit can result in cellular hypoplasia and precocious neurogenesis (66). Adult animal offspring that experienced developmental protein deficiency had fewer hippocampal progenitors, fewer neurons in the cornu ammonis 1 (CA1), and a reduced pyramidal layer volume (48,67,68). Reduced levels of BDNF (brain-derived neurotrophic factor) and IGF-1 (insulin-like growth factor 1), molecules that stimulate neuronal differentiation and survival, could underlie these observations (67). Pérez-García *et al.* showed that adult rats that experienced perinatal protein restriction and displayed these molecular and cellular alterations in the hippocampus underperformed on memorization tasks (69). Interestingly, a recent study showed that perinatal protein restriction in mice had transgenerational effects, impairing social motivation and recognition memory in F1 and F2 generations. The authors found reduced glucose metabolism in the prefrontal cortex and identified 21 autism-associated genes in an overall downregulated transcriptome (70).

Finally, fetal malnutrition may also contribute to the growing incidence of obesity and type 2 diabetes (71). Beyond a sedentary lifestyle and an imbalanced diet, increased susceptibility to metabolic diseases could also result from fetal misprogramming of the brain network that governs appetite, reward, and energy homeostasis (72). The hypothalamus is the central integrator of hunger and satiety signals to control appetite, relying on interconnected nuclei that sense leptin and gut peptides. Two neuronal populations in the arcuate nucleus play pivotal roles: the orexigenic AgRP (agouti-related peptide)/NPY (neuropeptide Y) neurons, inhibited by leptin, and the anorexigenic pro-opiomelanocortin (POMC) neurons, activated by leptin to produce α-melanocyte-stimulating hormone (αMSH). Binding to MC3/4 receptors in the paraventricular nucleus suppresses food intake and increases energy expenditure (73).

**Figure 2:**
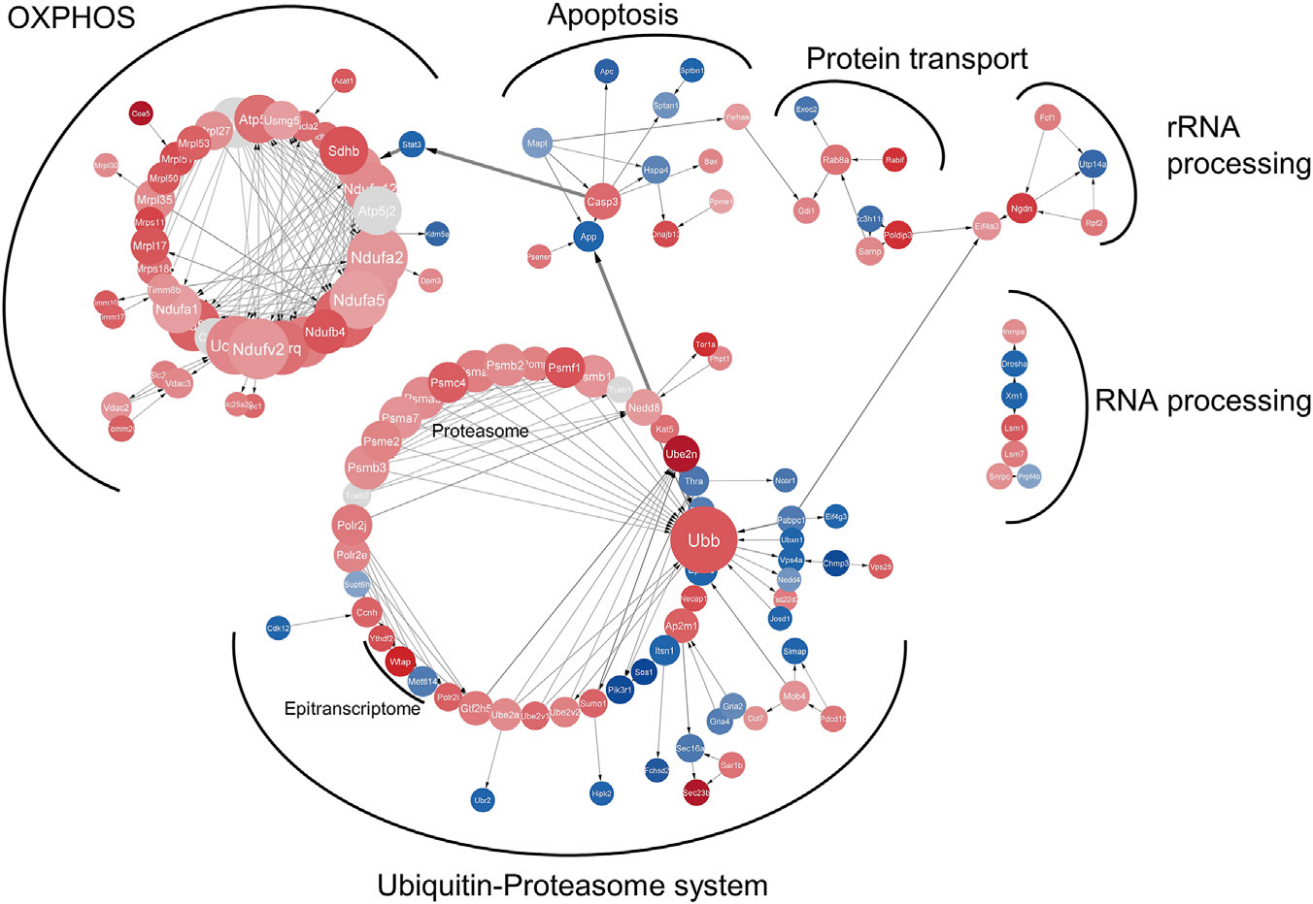
Network analysis of bulk RNA sequencing data from hypothalami dissected from G17 fetal rats exposed to an 8% protein-restricted diet during gestation. Utilizing CytoScape version 3.9.1 against the *Rattus norvegicus* reference genome (stringency of 0.80), the full STRING network was plotted. Employing STRING functional enrichment, 361 dysregulated genes were categorized into 6 major clusters (red: upregulated, blue: downregulated). Within the cell metabolism cluster, proteasome components were upregulated and linked to the ubiquitin hub, overseeing protein transfer to the proteasome. Concurrently, most genes governing mitochondrial OXPHOS exhibited upregulation, consistent with previous Western blot findings. The upregulation of STAT3, known for releasing the suppression of proapoptotic genes like caspase-3 in cancer cells (124), suggests analogous molecular mechanisms during brain development [dataset from (83)]. G, gestational day; OXPHOS, oxidative phosphorylation; rRNA, ribosomal RNA; STAT3, signal transducer and activator of transcription 3.

A crucial step in hypothalamic development unfolds early in life. In rodents, approximately half of the arcuate nucleus–POMC neurons at gestational day 13 (G13) transdifferentiate to generate the NPY neurons in the subsequent days (74). Gestational protein restriction disrupts this process of neurogenesis and cell fate determination (75,76), leading to fewer NPY neurons and attenuated projections from the arcuate nucleus to the paraventricular nucleus (77, 78, 79). This is manifested in hyperinsulinemia, increased body weight, hyperleptinemia, and altered leptin responsiveness, which are hallmarks of metabolic syndrome, in adult offspring (78,80,81). Another study found that altered expression of genes encoding NPY, POMC, and leptin receptor isoforms was evident in the protein-deprived fetal brain as early as G12 (82). Employing RNA sequencing, our research group found that gestational protein restriction led to dysregulation in 6.5% of the total number of detected genes in the hypothalamus of G17 rats (83). Network analysis assigned these genes to cellular metabolism, RNA processing, oxidative phosphorylation, and apoptosis (Figure 2).

## The Role of Sex and Timing

Female animals have often been excluded from preclinical studies due to the challenges posed by hormonal fluctuations associated with the menstrual cycle, which introduce increased variability and confounding results. However, their inclusion offers the opportunity to explore the etiology of sex-specific differences in neuroanatomy and behavior in human cohorts comprised of individuals who experienced the same conditions of dietary restriction (64). For example, men exposed to starvation in early pregnancy had smaller brain volumes and a higher likelihood of developing affective psychosis, whereas women did not (84,85). Some rodent studies have described sex-specific effects of fetal protein deficiency on neuroanatomy and behavior (Table 2). Hippocampal 5-HT_1A_ receptor dysfunction was exclusive to females (59), and malnourished females exhibited higher brain synaptic density (86). Females’ performance on stress tests was poorer than that of males (87), and females showed increased behavioral despair (46,50). In contrast, Nätt *et al.* discovered that male mice subjected to perinatal malnutrition showed more anxiety-like behavior than females (52). Transcriptome analysis of the prefrontal cortices of mice exposed to perinatal protein deficiency revealed numerous dysregulated genes that were not shared between the sexes despite similar behavioral phenotypes (70). Other studies have shown comparable responses in both sexes.

Thus, it remains inconclusive whether fetal protein deficiency affects brain development differently in males and females and the extent to which it may contribute to exacerbated neurocognitive impairments in either sex. The effects may be confined to specific brain regions or behaviors. Potential influencing factors include sex hormones, discordant neurodevelopmental timing, epigenetics, and divergent coping mechanisms (52,88). Additionally, discrepancies that arise from the use of different strains and species cannot be disregarded. A multiomics analysis revealed substantial differences in the molecular and behavioral responses to the same protein-restricted diet among 3 adult mouse strains (89).

In terms of timing, a noteworthy finding was that individuals who endured either the Dutch or the Chinese famine during the first trimester of pregnancy had a higher likelihood of suffering from schizophrenia (3, 4, 5). This suggests that the earliest stages of neurogenesis respond negatively to protein deprivation or that transient protein deficiencies, however brief, continue to impact amino acid supply even after protein uptake returns to normal. This raises the question of the time required for recovery from preconceptional amino acid deficiency, particularly if expectant mothers only transition to a protein-rich diet after pregnancy has been confirmed—often several weeks after conception—and therefore how long a fetus could be exposed to the delayed effects of deficiency. The importance of preconceptional nutrition in healthy infant development is exemplified by the necessity of supplementing with folic acid to prevent neural tube defects (90); commencing 1 month after conception is already past the period of most beneficial effects. At least one human cohort study reported that individuals exposed to the Dutch famine preconceptionally had smaller total brain volumes than their unexposed counterparts (85). It would certainly be interesting to investigate the effects of preconceptional protein deficiency in animal models.

That also prompts the question of how prolonged a protein deficiency must be to exert detrimental effects on brain development. Even a brief exposure to a low-protein diet limited to the first 3.5 days of gestation—the preimplantation period—sufficed to induce hyperactive and anxiety-like behavior as well as short-term memory deficits in adult mouse offspring (87,91). This remarkably short period of protein restriction reduced BCAA levels in blastocysts and blocked glucose metabolism, the primary energy source at this stage. In embryonic stem cells cultured from G3.5 blastocysts, MAPK (mitogen-activated protein kinase), ERK1/2 (extracellular signal-regulated kinase), and mTORC1 (mechanistic target of rapamycin complex 1) signaling pathways were dysregulated (92,93). Cell-tracing and immunohistochemical studies up to G12 showed reduced progenitor proliferation coupled with increased neuronal differentiation in the basal ganglia and cortex. This shift occurred without compensatory apoptosis, resulting in thicker cortices (91).

These findings emphasize the importance of maintaining adequate amino acid levels from the very onset of neurodevelopment and even preceding conception, challenging the conventional notion that adequate protein intake is only crucial later in pregnancy.

## Molecular Mechanisms

**Figure 3:**
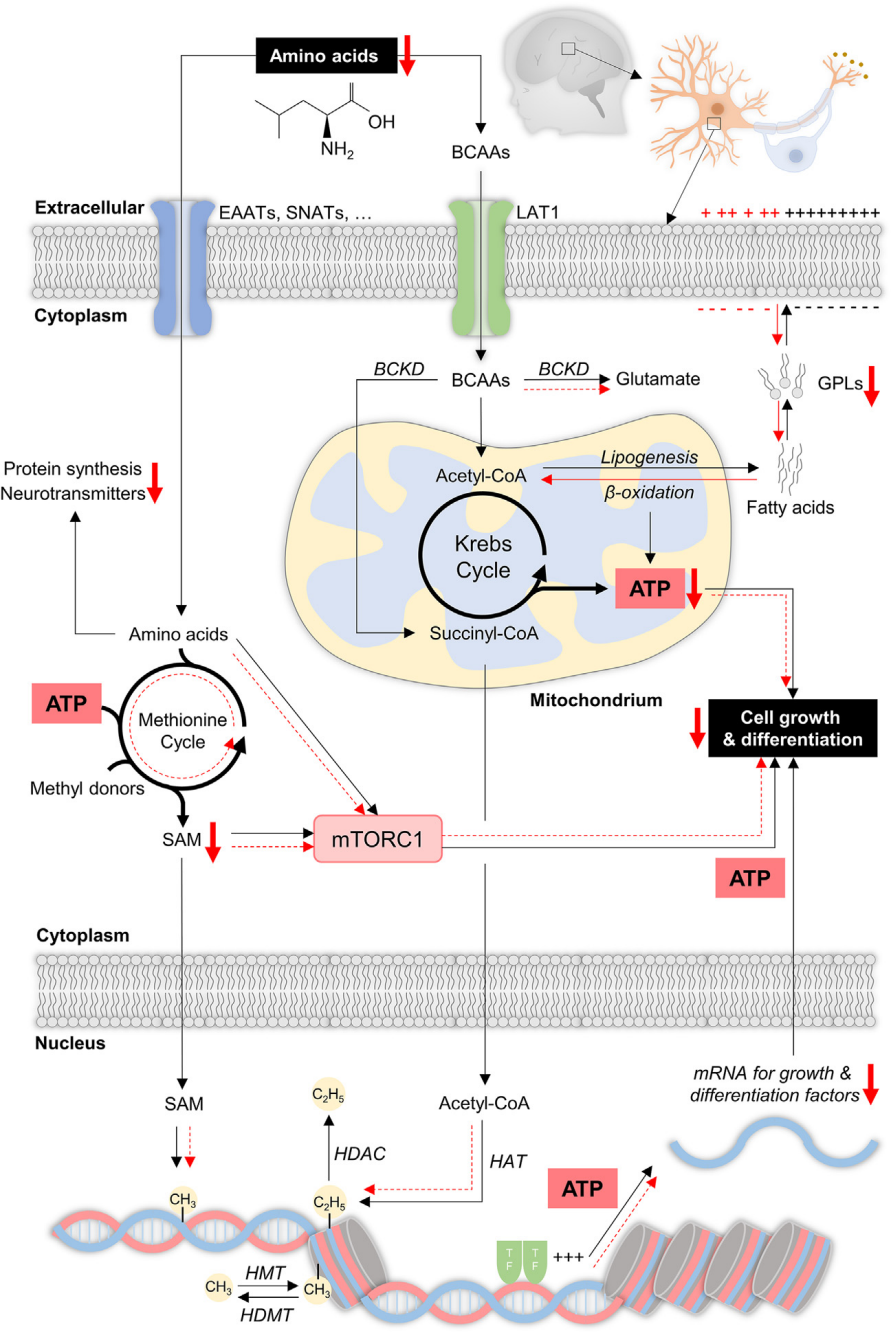
Concise overview of amino acid roles in neural precursor cell metabolism. Black lines denote physiological pathways, while red arrows indicate dysfunction upon cellular amino acid deprivation. LAT1 plays a vital role in BCAA uptake by neural precursors during development. This influences the growth, differentiation capacity, survival, and proper function of neural progenitors in the developing brain. ATP, adenosine triphosphate; BCAA, branched-chain amino acid; BCKD, branched-chain α-ketoacid dehydrogenase; EAAT, excitatory amino acid transporter; GPL, glycerophospholipids; HAT, histone acetyl transferase; HDAC, histone deacetylase; HDMT, histone dimethyl transferase; HMT, histone methyl transferase; LAT1, L-type amino acid transporter 1; mTORC1, mechanistic target of rapamycin complex 1; SAM, S-adenosyl methionine; SNAT, sodium-coupled neutral amino acid transporter; TF, transcription factor.

What are the underlying molecular mechanisms by which amino acid deficiency disrupts neurodevelopment? A longstanding assumption posits that endocrine signals, which mirror maternal physiology and external conditions, traverse the placenta to reach the fetus, where they modify epigenetic marks, activating or silencing genes and thereby influencing cellular programs and the structural blueprint of the brain (29). Enzymes responsible for establishing and erasing epigenetic marks rely on substrates and cofactors such as NAD, FAD, acetyl-CoA, vitamins, and SAM (S-adenosylmethionine), which are derived from nutrients and their metabolites (94,95). The best-known is the one-carbon metabolic pathway and involves methyl donors like folate, betaine, choline, and methionine, providing methyl groups to SAM—the universal methyl donor for DNA and histones (96) (Figure 3). Alternative methyl donors include amino acids such as serine, glycine, and histidine (97). Data on how fetal amino acid deficiency reshapes the cellular epigenome and influences neurodevelopmental pathways remain fragmented, but epigenetic end points are increasingly being incorporated into research studies.

We reported that supplementing the diet of pregnant rats with methyl donors increased hippocampal neural stem cell proliferation in offspring but not when combined with protein restriction, suggesting that both amino acids and micronutrient-derived methyl donors are crucial for hippocampal neurogenesis (98). Vucetic *et al.* demonstrated that the reduction in dopaminergic neurons following perinatal protein restriction in mice resulted from hypomethylation and subsequent overexpression of the cyclin-dependent kinase inhibitor 1c (62). This prevented neural progenitors from exiting the cell cycle, thereby providing a mechanistic clue to the etiology of an altered dopaminergic circuitry in neuropsychiatric disorders such as schizophrenia. In another study, the absence of anxiety-like behavior in female mice, compared with males fed a low-protein diet during development, was suggested to be due to increased methylation of the *Npy1r* gene in the female amygdala. This maintained *Npy1r* expression despite elevated *Egr1* levels, which suppressed *Npy1r* in males (52). Reduced brain *Bdnf* expression in neonatal rats exposed to a low-protein diet prenatally was found to be a consequence of repressive histone methylation (99). In the hypothalamus, we identified a hypomethylated *Pomc* promoter following perinatal protein restriction, which potentially contributes to altered feeding behavior in young rats (78). Interestingly, this dietary regimen also reduced levels of the epitranscriptomic mark *N*6-methyladenosine in the fetal hypothalamus, thus potentially affecting messenger RNA stability and turnover (83) (Figure 2). The involvement of the epitranscriptome in the mechanistic origins of metabolic disease is an emerging area of investigation (100).

Given the striking parallels with the effects of maternal psychosocial stress on offspring cognitive and neuropsychological outcomes (59,101), potential targets could also be retrieved from gene databases derived from these studies. For example, increased methylation of the glucocorticoid receptor gene *NR3C1* in adult offspring exposed to maternal psychological stress appears to be a key mechanism through which perinatal stress leaves a lasting mark (29,102). This mechanism may explain HPA axis dysregulation in protein deficiency.

In tandem with epigenetic mechanisms that modify gene expression, amino acids interact with cellular metabolism (103). The increased influx of amino acids is sensed by mTORC1 (104), the principal modulator of cell growth and differentiation (105). This activation induces a conformational change and the recruitment of cofactors, initiating RNA translation and activating essential signaling pathways. To meet the increased energy demands, a neural progenitor cell undergoes mitochondrial biogenesis and a metabolic shift from glycolysis to oxidative phosphorylation to upscale ATP (adenosine triphosphate) production (106,107). BCAAs and their metabolites thereby serve as substrates for acetyl-CoA and succinyl-CoA to fuel the Krebs cycle (Figure 3).

Recently, Knaus *et al.* (108) obtained important clues as to how cellular BCAA deficiency affects neuronal development through these molecular pathways and causes neurological disease. They had previously identified patients who exhibited microcephaly and autism spectrum disorder who harbor mutations in the *SLC7A5* gene. This gene encodes the LAT1 (L-type amino acid transporter 1) (109), prominently expressed in the blood-brain barrier and in neural cells (110,111). Its pivotal role in blood-brain barrier–dependent amino acid uptake was confirmed in mice lacking SLC7A5 in endothelial cells (109). However, selectively deleting *Slc7a5* in cerebrocortical neurons of perinatal mice revealed that cellular amino acid uptake is equally essential for neuronal maturation (108). The resulting deficiency of BCAAs failed to drive ATP generation, which is crucial for promoting neuroblast differentiation (106). To compensate, lipid catabolism increased, and fatty acids derived from triacylglycerols served as an alternative fuel source for the Krebs cycle. This shift reduced their availability for membrane glycerophospholipid production, which led to cortical neuroarchitectural defects (108). This phenotype is consistent with the observed 2-fold reduction in brain lipids, including ω-3 polyunsaturated fatty acids, in G19 rat fetuses exposed to a low-protein diet (45). Consequently, utilizing lipids for energy production to counteract amino acid deficiency means that they are no longer available for other processes dependent on them, such as cell membrane formation and myelinogenesis. This dual impact may exacerbate the adverse effects of protein-poor diets on brain development.

## Conclusions and Perspectives

Protein deficiency, whether resulting from compromised placental amino acid transport or malnutrition, represents a prevalent concern in pregnancy and neonatal care because it potentially impedes optimal neurocognitive development during infancy. Insights obtained from rodent studies suggest that misconfigured neuroendocrine axes and persistent alterations in neuroanatomy contribute to dysfunction of brain regions that have been implicated in cognitive abilities. Specific outcomes are contingent upon the severity and duration of the insult and could vary as a function of the asynchronous development of different brain regions and their distinct amino acid and energy requirements, as well as be influenced by genetic predisposition and external factors like infection (112). Fetal protein deficiency may also impact mental health in the long-term, mirroring the increased susceptibility to neuropsychiatric disorders in adults who experienced famine during prenatal life. However, while animal data hint at such a connection, direct, concrete evidence in humans is missing.

Rodent studies, while informative, warrant caution in extrapolating complex behavioral traits to humans. In addition, studies of protein deficiency tend to center on a single anatomical brain region, typically the hippocampus, to explain specific behavioral changes. Such one-to-one relationships seem improbable. Rather, anxiety, impaired learning, memory deficits, and altered reward-seeking behavior likely represent distinct phenotypic outcomes that arise from disrupted neural networks spanning multiple brain regions and their intercommunication. At the cellular level, abnormalities may manifest quantitatively (e.g., cell types, cell numbers, synapses, dendrites) or functionally (e.g., impaired neurotransmission, receptor dysfunction, interneuron types, excitation-inhibition imbalance). Presumably, protein deficiency impacts most, if not all, of these processes. At the molecular level, epigenetics and cellular metabolism emerge as primary pathways through which protein deficiency imprints lasting effects on brain development. While a wealth of data exist for the neuronal component, our understanding of its impact on other cell types such as oligodendrocytes, astrocytes, and microglia remains limited. To our knowledge, only one study has revealed that gestational protein restriction in rats induces microglia to a state of hyperactivity, elevating oxidative stress and neuroinflammatory markers in adult stages (113). In addition, exploring the role of the placenta as a fetal caretaker in abnormal nutritional conditions deserves more attention (114,115).

The challenge ahead is to untangle the interrelationships among these anomalies to gain a holistic view of how fetal protein deficiency ultimately affects human intellectual capacities and behavior. This pursuit includes deciphering the extent to which these adverse effects may or may not be reversible and identifying the optimal timing for interventions. While inconclusive, the potential of citrulline and arginine to stimulate fetal growth in the case of IUGR is promising (116, 117, 118) and may serve as an example of a simple but effective therapy. However, the assessment of its beneficial effects on neurodevelopment and behavior later in life requires further exploration and evaluation.

One of the solutions to enhance our understanding of how amino acids affect the molecular machinery of the cell, epigenome, (epi)transcriptome, and developmental pathways can be achieved by the use of multiomics technologies at the single-cell level across various brain regions. Employing cell-targeted genetics will play an important role in distinguishing direct and indirect effects. The integration of neurobehavioral end points into mammalian models that better match the timing of neurodevelopmental events in human gestation, such as nonhuman primates, will contribute to bridging the gap between rodents and humans. In experimental studies conducted with baboons, maternal nutrient restriction yielded phenotypes remarkably similar to those observed in rodents subjected to a protein-restricted diet (Table 1). Innovative models like brain organoids offer the advantage of being able to test a diverse range of nutritional stressors on neurogenesis without interference from other environmental factors [e.g., (119)]. Additionally, exploration of rare genetic disorders related to amino acid metabolism may shed further light on the role of specific enzymes and the dependency of certain amino acids in neurodevelopmental processes (109,120,121). Moreover, it is imperative to thoroughly investigate how the timing of nutritional deficiencies and sex influence phenotypic outcomes.

In summary, this review underscores the critical role of adequate protein intake before and during pregnancy, as well as lactation, in supporting optimal fetal brain development and mitigating the risk of neurocognitive impairments in offspring. It emphasizes the importance of incorporating food frequency questionnaires into public health studies, a practice that has been largely overlooked, and underscores the importance of shaping uniformity in national dietary guidelines with regard to the consumption of plant-based diets during pregnancy (122).
